# Multiswarm Particle Swarm Optimization with Transfer of the Best Particle

**DOI:** 10.1155/2015/904713

**Published:** 2015-08-05

**Authors:** Xiao-peng Wei, Jian-xia Zhang, Dong-sheng Zhou, Qiang Zhang

**Affiliations:** ^1^School of Mechanical Engineering, Dalian University of Technology, Dalian 116024, China; ^2^Key Laboratory of Advanced Design and Intelligent Computing, Ministry of Education, Dalian University, Dalian 116622, China

## Abstract

We propose an improved algorithm, for a multiswarm particle swarm optimization with transfer of the best particle called BMPSO. In the proposed algorithm, we introduce parasitism into the standard particle swarm algorithm (PSO) in order to balance exploration and exploitation, as well as enhancing the capacity for global search to solve nonlinear optimization problems. First, the best particle guides other particles to prevent them from being trapped by local optima. We provide a detailed description of BMPSO. We also present a diversity analysis of the proposed BMPSO, which is explained based on the Sphere function. Finally, we tested the performance of the proposed algorithm with six standard test functions and an engineering problem. Compared with some other algorithms, the results showed that the proposed BMPSO performed better when applied to the test functions and the engineering problem. Furthermore, the proposed BMPSO can be applied to other nonlinear optimization problems.

## 1. Introduction

Many nonlinear optimization problems are attracting increasing attention from researchers, with conflicting objectives and using various random search methods. Global optimization algorithms are employed widely to solve these problems [1]. Particle swarm optimization (PSO) is a type of random optimization method, which was inspired by the flocking behavior of birds [2, 3]. Kennedy and Eberhart were the first to propose PSO [4]. Compared with other swarm intelligence algorithms, PSO has a simple structure and rapid convergence rate, and it is easy to perform, which makes it an effective method for solving nonlinear optimization problems [5, 6].

In recent years, many researchers have tried to improve PSO to overcome its shortcomings; that is, it exhibits premature convergence and is readily trapped by local optima [7]. In order to improve the efficiency and effectiveness of multiobjective particle swarm optimization, a competitive and cooperative coevolutionary multiobjective particle swarm optimization algorithm (CCPSO) was presented by Goh et al. in 2010 [8]. A competitive and cooperative coevolution mechanism was introduced in the proposed CCPSO, which does not handle the ZDT4 problem well, so it cannot be applied widely. Rathi and Vijay presented a modified PSO (EPSO) in 2010 [9], where two stages are used to balance the local and global search. First, the bandwidth of microstrip antenna (MSA) was modeled by a benchmark function. Second, the first output was then employed as an input to obtain the new output in the form of five parameters. The proposed EPSO is efficient and accurate. In 2011, a multiswarm self-adaptive and cooperative particle swarm optimization (MSCPSO) was proposed by Zhang and Ding [10], which employs four subswarms: subswarms 1 and 2 are basic, but subswarm 3 is influenced by subswarms 1 and 2, while subswarm 4 is affected by subswarms 1, 2, and 3. The four subswarms employ a cooperative strategy. While it achieved good performances in solving complex multimodal functions, MSCPSO was not applied to practical engineering problems. A new chaos-enhanced accelerated PSO algorithm was proposed by Gandomi et al. in 2013 [11], which delivered good performances when applied to a complex problem, but it is not easy to operate. Ding et al. developed the multiswarm cooperative chaos particle swarm optimization algorithm in 2013 [12], which includes chaos and multiswarm cooperative strategies. This method was proposed only to optimize the parameters of a least squares support vector machine. In order to establish and optimize the alternative path, an efficient routing recovery protocol with endocrine cooperative particle swarm optimization was proposed by Hu et al. in 2015 [13], which employs a multiswarm evolution equation. Qin et al. proposed a novel coevolutionary particle swarm optimizer with parasitic behavior in 2015 [14], where a host swarm and parasite swarm exchange information. This method performs better in terms of the solution accuracy and convergence, but the structure is complex.

In the present study, we introduce a multiswarm particle swarm optimization with transfer of the best particle (BMPSO), in order to improve the global search capacity and to avoid trapping in local optima. The proposed algorithm employs three slave swarms and a master swarm. The best particle and worst particle are selected by the PSO from every slave swarm. The best particle is then transferred to the next slave swarm to replace the worst particle. The three best particles are then stored in the master swarm. Finally, the optimal value is obtained by PSO from the master swarm. Compared with other optimization algorithms, this parasitism strategy is easier to understand. The control parameters for the proposed algorithm do not increase and it can find the optimal solution easily.

The remainder of this paper is organized as follows. In Section 2, we provide an overview of the standard PSO. Our proposed BMPSO is explained in Section 3. We present the results of our numerical experimental simulations as well as comparisons in Section 4. In Section 5, we apply our proposed BMPSO to a practical engineering problem. Finally, we give our conclusions.

## 2. Standard PSO

In 1995, the standard PSO algorithm was presented by Kennedy and Eberhart [4]. It was developed further subsequently due to its easy implementation, high precision, and fast convergence. Similar to other evolutionary algorithms, it starts from random solutions and performs iterative searches to find the best solution. The quality of the solution is evaluated based on fitness.

PSO utilizes a swarm population of no quality and volume particles. Each particle moves in a *D*-dimensional space according to its own experience and that of neighbors while searching for the best solution. The position of the *i*′th particle is expressed by the following vector: *x*_*i*_ = [*x*_*i*1_,…, *x*_*id*_]. The velocity is expressed by the following vector: *v*_*i*_ = [*v*_*i*1_,…, *v*_*id*_]. In each step, the particles move according to the following formulae:

(1) vijt+1=vijt+c1r1pij−xijt+c2r2pgj−xijt,

(2) xijt+1=xijt+vijt+1,

where *j* = 1,…, *d*, *c*_1_ and *c*_2_ are acceleration constants, *r*_1_ and *r*_2_ represent two independent uniformly distributed random variables between 0 and 1, *p*_*ij*_ is the best previous position of the particle itself, and *p*_*gj*_ is the best global value.

In 1998, an inertia weight *w* was introduced into formula (1) by Shi and Eberhart [15], as shown by the following formula [18]:

(3) vijt+1=wvijt+c1r1pij−xijt+c2r2pgj−xijt.

It was demonstrated that the inclusion of a suitable inertia weight *w* allows the best solution to be searched more accurately. This weight can balance exploration and exploitation.

The standard PSO procedure is illustrated as follows.

**Step 1 .**

Initialize the position and velocity of each particle randomly in the population.

**Step 2 .**

Evaluate the value of the fitness function. Store the values in pbest and gbest.

**Step 3 .**

Update the position and velocity of each particle using formulae (2) and (3).

**Step 4 .**

Compare pbest and gbest, and then update gbest.

**Step 5 .**

Assess whether the conditions are met or not. If not, go back to Step 3.

## 3. General Description of BMPSO

The standard PSO is readily trapped by local optima and it is susceptible to premature convergence [19], while solving multiobjective problems with constraint conditions. Thus, we propose a new method for multiswarm PSO with transfer of the best particle called BMPSO. Our proposed algorithm can be applied to unconstrained problems but also with constraint conditions. It has the ability to escape local optima and prevent premature convergence.

### 3.1. Best Particle Coevolutionary Mechanism

**Figure 1:**
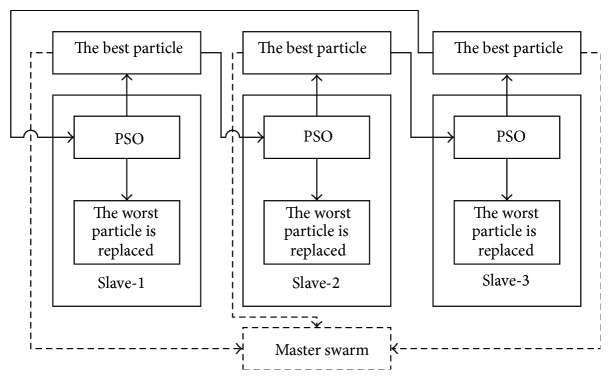
Structure of BMPSO.

In this section, we provide a general description of BMPSO. The proposed method employs three slave swarms and a master swarm. There is a specific relationship among the four swarms. We propose three swarms as slaves, which are designated slave-1, slave-2, and slave-3. First, the best particle-1 of slave-1 is selected, which is then stored in the master swarm. Second, the worst particle-2 from slave-2 is replaced with the best particle-1. The best particle-2 is then found, which is stored in the same manner as particle-1. The same strategy is applied to slave-3. When the three slave swarms have been processed, the master best particle is found in the master swarm as the optimal value. This strategy is not complete until various conditions have been met. The proposed BMPSO involves cooperation and competition. This evolutionary strategy is called parasitism in nature [20]. The structure of the BMPSO is shown in Figure 1.

Our improved algorithm comprises three slave swarms and a master swarm, where a parasitism strategy is used to balance exploration and exploitation. The control parameters for the proposed BMPSO include the number of particles, inertia weight, dimension of particles, acceleration coefficients, and iterations. The number of particles is determined by the complexity of the problem, that is, from 5 to 100. The inertia weight decides how to inherit from current velocity. The dimension of the particles is determined by the optimization problem as the dimension of the result required. The acceleration coefficients give the particles the capacity for self-summary and learning from others, and they usually have a value of 2. The number of iterations can be determined by the experimental requirements. The pseudocodes for the BMPSO are presented as follows.

*Algorithm BMPSO*

 
**Begin**
 Specify the population of each slave swarm
 Initialize the velocity and position
 Evaluate the fitness value
 Find the best particle and worst particle in each slave swarm
 Use the best particle to replace the worst particle in the next slave swarm
 Store the best particle in the master swarm
 Find the optimal value in the master swarm
 
**Repeat**
 
**Until** a terminate-condition is met
 
**End**

### 3.2. Diversity Analysis of BMPSO

**Figure 2:**
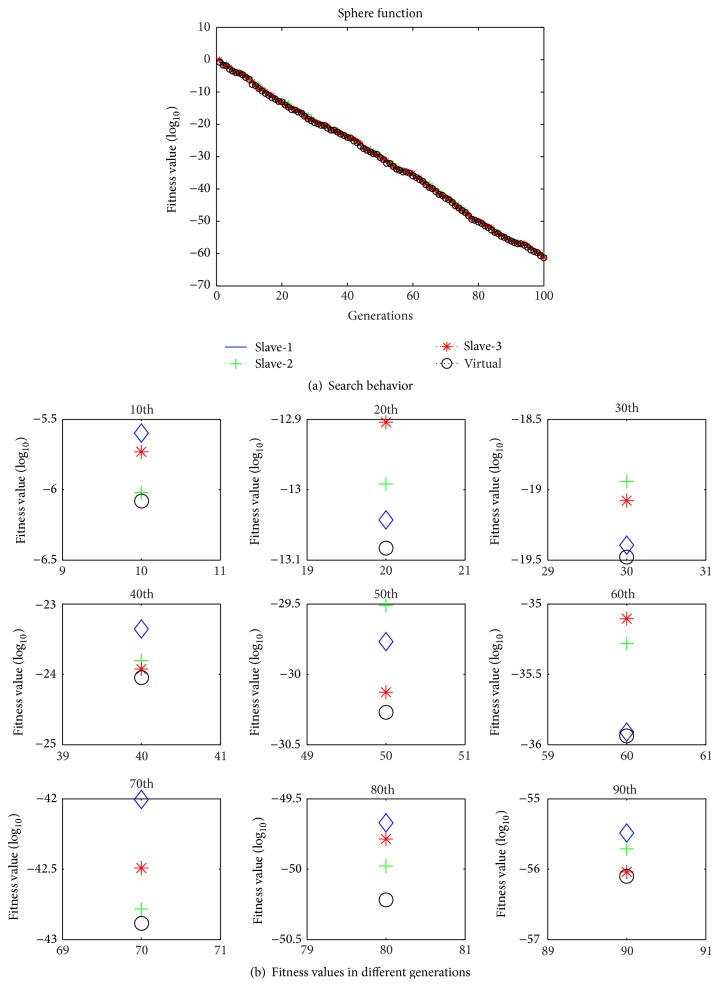
Evolutionary processes based on the Sphere function.

In order to explain the proposed algorithm in detail, we illustrate the search capacity of each particle in the four swarms. The worst particle is replaced by the best particle. The best particle will lead the other particles away from a local optimum.  Figure 2 shows the evolutionary processes for the particles in slave-1, slave-2, slave-3, and the master swarm.

The evolutionary processes based on the Sphere function in 20*D* are shown in Figure 2. The graphs represent the results obtained by the proposed algorithm in a single run.  Figure 2(a) shows that the particles in the four swarms performed their search behavior in a smooth manner. The diversity was improved when the best particle replaced the worst particle. This point is illustrated clearly by the experiments with the test function.  Figure 2(b) shows the status of the particles during different generations based on the distances among four particles. The proposed algorithm made a greater effort to avoid becoming trapped by a local optimum after the 50th generation, while still considering the convergence speed. Thus, it maintained the diversity of the fitness value but not at the cost of the convergence speed.

**Figure 3:**
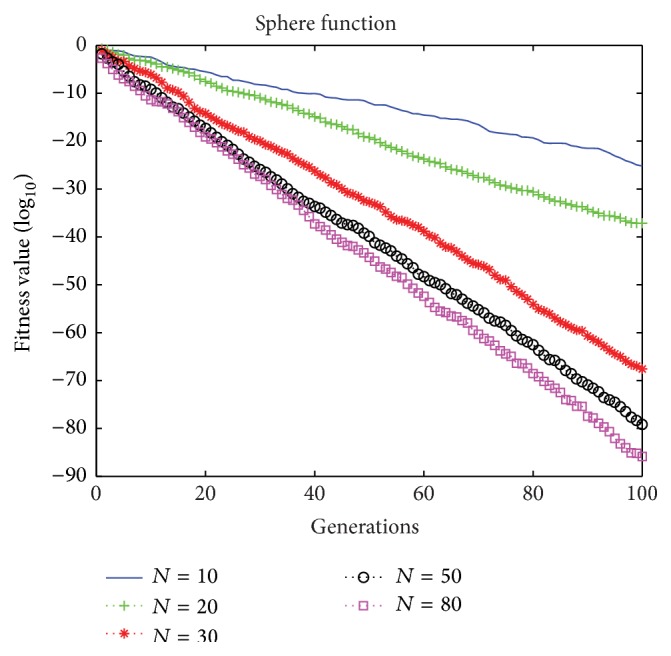
Convergence characteristics for the Sphere function.

The number of particles affects the optimization ability of BMPSO. In order to verify this, Figure 3 illustrates the convergence characteristics using the Sphere function as in the example (*N* = 10, 20, 30, 50, 80).

Figure 3 shows that the final optimized fitness value tended to improve as the number of particles increased. However, this is not as obvious in the later stage. In addition, there must be a greater communication cost when the number of particles increases. In the proposed BMPSO, *N* = 10–30 is sufficient for most problems.

**Figure 4:**
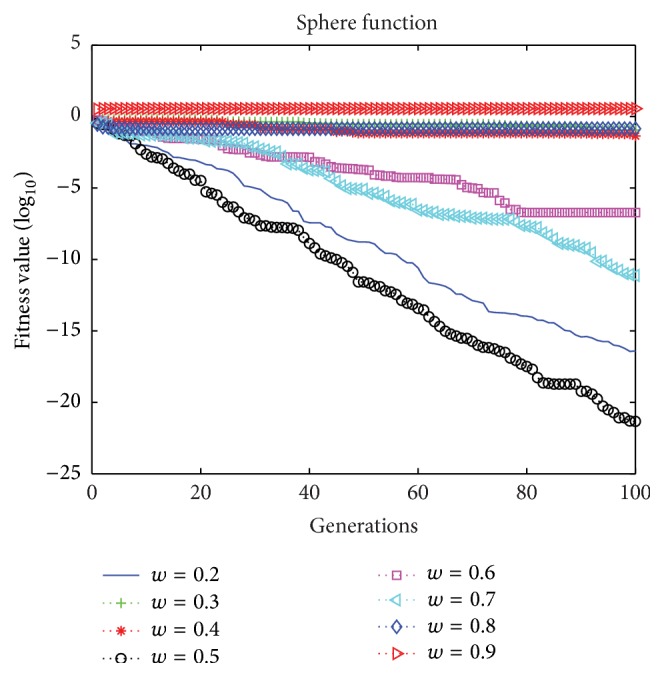
Different inertia weights.

In the standard PSO, the inertia weight *w* is very important, because it affects the balance between local and global search. It describes the influence of inertia on speed. When the inertia weight *w* is higher, the global optimization ability is better. In order to determine the appropriate value for *w*, we performed experiments with the test function and Figure 4 shows the results obtained with different values for *w*.

Figure 4 clearly demonstrates that the optimum fitness value is not easy to reach, when the inertia weight *w* is too small or too large. In the proposed BMPSO, *w* = 0.5 is the optimum value.

**Figure 5:**
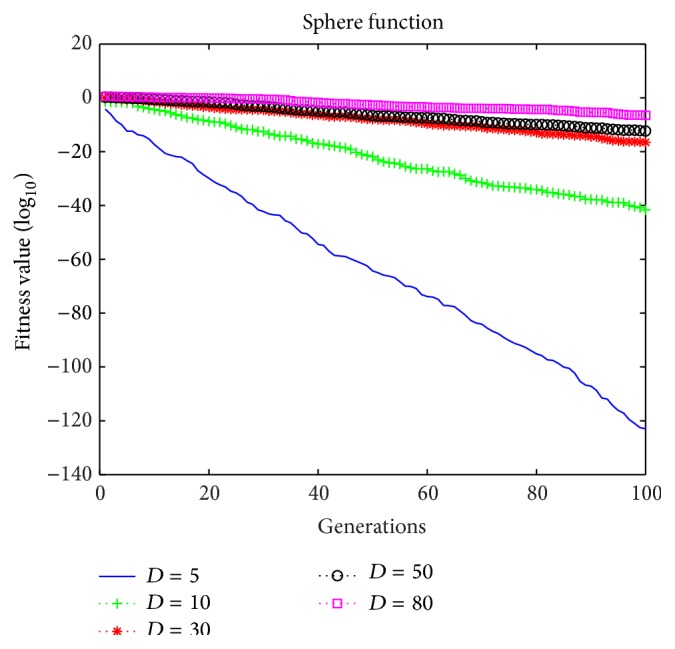
Results obtained using different dimensions (*D*).

To a certain extent, the dimension (*D*) of particles represents the complexity of a problem, where the search capacity decreases as *D* increases. We performed experiments to determine the suitable scope for *D* using the Sphere function as an example, and Figure 5 illustrates the results obtained.

Figure 5 shows that the proposed BMPSO is more effective with a smaller dimension. In the proposed algorithm, *D* = 5–30 is a suitable dimension.

In this section, we considered the influence of different parameters on the proposed algorithm. This diversity analysis demonstrated the effectiveness of BMPSO, where the best particle shares more information with others and it replaces the worst particle during the evolutionary process. This guides the other particles to prevent them from being trapped by local optima and avoids premature convergence. The proposed algorithm balances exploration and exploitation in an effective manner.

## 4. Numerical Experiments

In order to determine whether the proposed algorithm is effective for nonlinear optimization problems, we performed experiments using standard test functions [21, 22]. The proposed algorithm was simulated and verified on the MATLAB platform. The results were compared with those obtained using a modified particle swarm optimizer called W-PSO [15], an improved particle swarm optimization combined with chaos called CPSO [16], a completely derandomized self-adaptation in evolution strategies called CMAES [17], and a multiswarm self-adaptive and cooperative particle swarm optimization called MSCPSO [10].

### 4.1. Standard Test Functions

In order to validate the efficiency of BMPSO, the six standard test functions were employed in experiments to search for the optimum value of the fitness function. The six test functions were Sphere, Rastrigin, Griewank, Schwefel, Elliptic, and Rosenbrock [23]. The global optima for these six standard test functions are equal to zero. The formulae for the six functions are shown in Table 1.

Sphere, Schwefel, and Elliptic are typical unimodal functions, and thus it is relatively easy to search for the optimum value, which is considered to be the simple single mode state. Rastrigin and Griewank are typical nonlinear multimodal functions, with a wide search space. The peak shape emerges in high and low volatile hops, and it is usually considered difficult to handle complex multimodal problems using optimization algorithms. The global optimum value for Rosenbrock is in a smooth and narrow parabolic valley. Thus, it is difficult to search for the optimum value because the function supplies little information for optimization algorithms. The six standard test functions comprise a class of complex nonlinear problems [24].

### 4.2. Results and Comparative Study

In order to compare the results in a standard manner, all of the experiments were performed on the same computer and using MATLAB R2013b. The parameters for the algorithms included the number of particles *N*, inertia weight *w*, dimension of particles *D*, acceleration coefficients *c*_1_ and *c*_2_, and iterations *M*, which were set as follows. For W-PSO and the proposed BMPSO, the parameters were set as *N* = 30, *c*_1_ = *c*_2_ = 2, *w* = 0.5, *M* = 1000, and *D* = 10, 30. For CPSO, the parameters were set as *N* = 30, *c*_1_ = *c*_2_ = 2, *w*_max_ = 0.9, *w*_min_ = 0.4, *M* = 1000, *x*_max_ = 10^*∗*^ones(1, *D*),  *x*_min_ = −10^*∗*^ones(1, *D*), and *D* = 10, 30. For CMAES, the parameters were set as described in [16]. For MSCPSO, the parameters were *N* = 30, *c*_1_ = *c*_2_ = 2, *w*_max_ = 0.9, *w*_min_ = 0.4, *M* = 1000, *D* = 10, 30, *a*_1_ = 1/6, *a*_2_ = 1/3, and *a*_3_ = 1/2. In this study, all of the experiments using the algorithms were replicated independently 50 times. The best, worst, mean, and standard deviations of the fitness values were recorded, so the results summarized the performance of the algorithms [25]. The simulation results are presented in Table 2 (*D* = 10) and Table 3 (*D* = 30).

In Tables 2 and 3, the best results are highlighted in bold. Table 2 shows that the proposed BMPSO performed better compared with W-PSO, CPSO, CMAES, and MSCPSO for Rosenbrock on “Std.” The results obtained by BMPSO were closest to the theoretical value. It should be noted that the optimal value of 0 could be found for Rosenbrock. Table 3 shows that our proposed BMPSO performed better for Sphere, Griewank, Schwefel, Elliptic, and Rosenbrock compared with the other algorithms, whereas W-PSO performed the best for Rastrigin. According to this analysis, we conclude that the proposed BMPSO has a greater capacity for handling most nonlinear optimization problems compared with W-PSO, CPSO, CMAES, and MSCPSO.

## 5. Engineering Application

**Figure 6:**
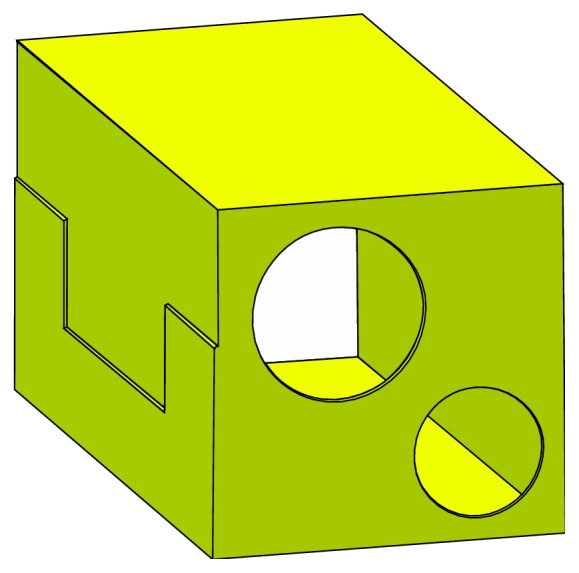
A 3*D* model of a gearbox.

We also solved an engineering problem based on lightweight optimization design for a gearbox to confirm the efficiency of the proposed BMPSO. In the development of the autoindustry, lightweight optimization design for gearboxes is attracting much attention because of energy and safety issues. There are three standard types of lightweight methods [26, 27]. In order to solve the problem effectively, Yang et al. proposed a method that establishes a simplified model first, before analyzing it using ANSYS and then establishing an approximate model, with the response surface methodology, followed by final optimization with a genetic algorithm (GA) [28]. A simplified 3*D* model of a gearbox is shown in Figure 6. This simplified principle has no effect on the finite element analysis.

In the optimization process, the variables are the bottom thickness *x*_1_, axial thickness *x*_2_, and lateral thickness *x*_3_. Detailed descriptions of the establishment of the fitness function can be found in [28]. The optimization problem for the lightweight optimization design of a gearbox can be represented by the following formula:

(4) min⁡fx1,x2,x3,fx1,x2,x3=4.6896+3.3676∗x1+0.5282∗x2+1.0110∗x3,87.2571+24.4741∗x1−1.6680∗x2+5.1004∗x3>120,0.1715−0.0121∗x1−0.0011∗x2−0.0026∗x3<0.09,73.1417−3.7565∗x1+0.0754∗x2−1.0626∗x3<200,x1∈3,6,  x2∈14,20,  x3∈3,8.

For the proposed BMPSO, the parameters were set as follows: *N* = 30, *c*_1_ = *c*_2_ = 2, *w* = 0.5, *M* = 1000, and *D* = 3. The parameters for the GA were set as described in [28]. This experiment was performed with 50 independent runs. The results obtained were expressed as the average of 50 runs.

The optimization results presented in Table 3 show that the weight of the gearbox is 31.3458 kg according to the proposed BMPSO, which is 8.4682 kg less than the original and 0.6368 kg less compared with GA. Thus, it can be concluded that the proposed BMPSO performed better compared with the GA. Therefore, the proposed BMPSO is effective in handling complex problems (see Table 4).

## 6. Conclusions

In this study, we proposed an improved multiswarm PSO method with transfer of the best particle called BMPSO, which utilizes three slave swarms and a master swarm that share information among themselves via the best particle. All of the particles in the slave swarms search for the global optimum using the standard PSO. The best particle replaces the worst in order to guide other particles to prevent them from becoming trapped by local optima. We performed a diversity analysis of BMPSO using the Sphere function. We also applied the proposed algorithm to standard test functions and an engineering problem.

We introduced parasitism into the standard PSO to develop a multiswarm PSO that balances exploration and exploitation. The advantages of the proposed BMPSO are that it is easy to understand, has a low number of parameters, and is simple to operate. In the proposed algorithm, the strategy of using the best particle to replace the worst enhances the global search capacity when solving nonlinear optimization problems. The optimum solution is obtained from the master swarm. The diversity analysis also demonstrated the obvious improvements obtained using the proposed algorithm. Compared with previously proposed algorithms, our BMPSO delivered better performance. The result obtained by the BMPSO in an engineering problem demonstrated its efficiency in handing a complex problem.

In further research, convergence analysis of the method should be performed in detail. Our further research will also focus on extensive evaluations of the proposed BMPSO by solving more complex and discrete practical optimization problems.

## Figures and Tables

**Table 1:** Benchmark functions.

	Test function	Search range	*f*_min⁡_
Sphere	$f_{1}(x)=\overset{D}{\underset{i=1}{\sum }}x_{i}^{2}$	[−100,100]^*D*^	0

Rastrigin	$f_{2}(x)=\overset{D}{\underset{i=1}{\sum }}\left[x_{i}^{2}-10\cos\left(2\pi x_{i}\right)+10\right]$	[−10,10]^*D*^	0

Griewank	$f_{3}\left(x\right)=\frac{1}{4000}\overset{D}{\underset{i=1}{\sum }}x_{i}^{2}-\prod_{i=1}^{D}\cos\left(\frac{x_{i}}{\sqrt{i}}\right)+1$	[−50,50]^*D*^	0

Schwefel	$f_{4}(x)=\overset{D}{\underset{i=1}{\sum }}\left|x_{i}\right|+\prod_{i=1}^{D}\left|x_{i}\right|$	[−10,10]^*D*^	0

Elliptic	$f_{5}(x)=\overset{D}{\underset{i=1}{\sum }}10^{6(i-1)/(D-1)}x_{i}^{2}$	[−100,100]^*D*^	0

Rosenbrock	$f_{6}(x)=\overset{D-1}{\underset{i=1}{\sum }}\left[100{\left(x_{i}^{2}-x_{i+1}\right)}^{2}+{\left(x_{i}-1\right)}^{2}\right]$	[−100,100]^*D*^	0

**Table 2:** Results for 10*D* problems.

Test functions	MSCPSO	W-PSO	CPSO	CMAES	BMPSO
	[10]	[15]	[16]	[17]	Present
*f*_1_					
Best	1.628*e* ^−001^	1.205*e* ^−005^	5.260*e* ^−002^	2.430*e* ^−002^	1.115**e** ^−132^
Worst	3.301*e* ^−000^	3.589*e* ^−002^	1.983*e* ^−000^	6.471*e* ^−001^	1.967**e** ^−121^
Mean	1.741*e* ^−000^	4.048*e* ^−003^	5.484*e* ^−001^	1.555*e* ^−001^	2.383**e** ^−122^
Std.	6.880*e* ^−001^	7.038*e* ^−003^	4.282*e* ^−001^	1.461*e* ^−001^	6.449**e** ^−122^

*f*_2_					
Best	3.263*e* ^+001^	2.011*e* ^−000^	8.603*e* ^−000^	4.358*e* ^+001^	**1.990** **e** ^−000^
Worst	6.669*e* ^+001^	1.696*e* ^+001^	4.498*e* ^+001^	1.212*e* ^+002^	**9.950** **e** ^−000^
Mean	5.471*e* ^+001^	7.750*e* ^−000^	2.432*e* ^+001^	8.182*e* ^+001^	**4.759** **e** ^−000^
Std.	7.737*e* ^−000^	3.677*e* ^−000^	7.737*e* ^−000^	1.596*e* ^+001^	**1.894** **e** ^−000^

*f*_3_					
Best	6.319*e* ^−002^	1.008*e* ^−006^	2.900*e* ^−003^	4.324*e* ^−000^	9.807**e** ^−008^
Worst	3.469*e* ^−001^	2.046*e* ^−003^	2.156*e* ^−001^	1.557*e* ^+001^	2.314**e** ^−004^
Mean	1.945*e* ^−001^	4.139*e* ^−004^	6.938*e* ^−002^	8.689*e* ^−000^	2.985**e** ^−005^
Std.	7.117*e* ^−002^	4.641*e* ^−004^	5.029*e* ^−002^	2.209*e* ^−000^	4.811**e** ^−005^

*f*_4_					
Best	2.331*e* ^−000^	7.700*e* ^−003^	3.759*e* ^−001^	5.834*e* ^−000^	8.807**e** ^−078^
Worst	4.909*e* ^−000^	5.131*e* ^−001^	3.616*e* ^−000^	1.992*e* ^+014^	5.982**e** ^−070^
Mean	3.749*e* ^−000^	1.415*e* ^−001^	1.650*e* ^−000^	1.399*e* ^+000^	2.574**e** ^−071^
Std.	5.918*e* ^−001^	1.053*e* ^−001^	6.698*e* ^−001^	4.010*e* ^+013^	9.904**e** ^−071^

*f*_5_					
Best	2.746*e* ^−000^	1.251*e* ^−000^	5.386*e* ^+001^	5.734*e* ^+005^	3.412**e** ^−133^
Worst	9.176*e* ^+003^	3.357*e* ^+002^	1.877*e* ^+004^	1.267*e* ^+007^	2.431**e** ^−117^
Mean	1.340*e* ^+003^	6.198*e* ^+001^	3.090*e* ^+003^	4.678*e* ^+006^	1.030**e** ^−118^
Std.	1.652*e* ^+003^	7.274*e* ^+001^	4.007*e* ^+003^	3.376*e* ^+006^	4.393**e** ^−118^

*f*_6_					
Best	7.326*e* ^−000^	5.662*e* ^−000^	1.184*e* ^+001^	1.785*e* ^+001^	**0**
Worst	6.411*e* ^+001^	1.274*e* ^+001^	1.620*e* ^+002^	4.574*e* ^+005^	8.595**e** ^−000^
Mean	1.783*e* ^+001^	8.486*e* ^−000^	6.249*e* ^+001^	1.993*e* ^+004^	5.465**e** ^−001^
Std.	1.319*e* ^+001^	1.206**e** ^−000^	3.946*e* ^+001^	6.735*e* ^+004^	1.704*e* ^−000^

**Table 3:** Results for 30*D* problems.

Test functions	MSCPSO	W-PSO	CPSO	CMAES	BMPSO
	[10]	[15]	[16]	[17]	Present
*f*_1_					
Best	8.344*e* ^−000^	2.803*e* ^−001^	2.582*e* ^−000^	1.643*e* ^+004^	1.490**e** ^−050^
Worst	1.766*e* ^+001^	1.486*e* ^−000^	1.481*e* ^+001^	3.302*e* ^+004^	4.029**e** ^−042^
Mean	1.377*e* ^+001^	8.158*e* ^−001^	9.139*e* ^+000^	2.448*e* ^+004^	1.279**e** ^−043^
Std.	1.904*e* ^−000^	2.639*e* ^−001^	2.710*e* ^−000^	4.240*e* ^+003^	5.823**e** ^−043^

*f*_2_					
Best	1.888*e* ^+002^	1.354**e** ^−000^	1.092*e* ^+002^	1.467*e* ^+004^	5.474*e* ^−000^
Worst	2.543*e* ^+002^	2.289**e** ^+001^	2.293*e* ^+002^	3.989*e* ^+004^	5.234*e* ^+001^
Mean	2.292*e* ^+002^	8.902**e** ^−000^	1.595*e* ^+002^	2.575*e* ^+004^	2.219*e* ^+001^
Std.	1.442*e* ^+001^	4.894**e** ^−000^	2.714*e* ^+001^	4.326*e* ^+003^	8.802*e* ^−000^

*f*_3_					
Best	4.156*e* ^−001^	1.200*e* ^−002^	9.400*e* ^−002^	2.881*e* ^+001^	3.600**e** ^−003^
Worst	8.146*e* ^−001^	8.402*e* ^−002^	7.377*e* ^−001^	4.071*e* ^+001^	1.715**e** ^−002^
Mean	6.618*e* ^−001^	3.750*e* ^−002^	4.080*e* ^−001^	3.416*e* ^+001^	8.690**e** ^−003^
Std.	9.181*e* ^−002^	1.379*e* ^−002^	1.088*e* ^−001^	2.636*e* ^−000^	2.938**e** ^−003^

*f*_4_					
Best	1.225*e* ^+001^	1.798*e* ^−000^	8.544*e* ^−000^	2.790*e* ^+032^	9.794**e** ^−034^
Worst	1.826*e* ^+001^	4.753*e* ^−000^	1.595*e* ^+001^	4.676*e* ^+045^	2.946**e** ^−029^
Mean	1.626*e* ^+001^	3.334*e* ^−000^	1.243*e* ^+001^	2.789*e* ^+044^	3.206**e** ^−030^
Std.	1.114*e* ^−000^	6.914*e* ^−001^	1.680*e* ^−000^	8.220*e* ^+044^	7.794**e** ^−030^

*f*_5_					
Best	1.584*e* ^+004^	1.892*e* ^+003^	1.649*e* ^+004^	1.875*e* ^+008^	1.657**e** ^−048^
Worst	4.792*e* ^+005^	1.983*e* ^+004^	2.789*e* ^+005^	1.363*e* ^+009^	4.245**e** ^−040^
Mean	2.103*e* ^+005^	6.978*e* ^+003^	1.269*e* ^+005^	7.935*e* ^+008^	2.546**e** ^−041^
Std.	1.185*e* ^+005^	3.620*e* ^+003^	6.848*e* ^+004^	2.416*e* ^+008^	8.401**e** ^−041^

*f*_6_					
Best	1.944*e* ^+002^	5.434*e* ^+001^	4.955*e* ^+002^	2.323*e* ^+009^	2.632**e** ^+001^
Worst	2.259*e* ^+003^	2.054*e* ^+002^	2.649*e* ^+003^	1.057*e* ^+010^	3.016**e** ^+001^
Mean	6.547*e* ^+002^	1.008*e* ^+002^	1.586*e* ^+003^	5.284*e* ^+009^	2.907**e** ^+001^
Std.	3.655*e* ^+002^	2.963*e* ^+001^	5.188*e* ^+002^	1.723*e* ^+009^	7.100**e** ^−001^

**Table 4:** Optimization results.

Method	*X*_1_ (mm)	*X*_2_ (mm)	*X*_3_ (mm)	*M* (kg)
Original	4	20	7	39.8140
GA	4.8182	14.0024	3.0029	31.9826
BMPSO	4.8185	14.0001	3.0017	31.3458
